# Rational Design of Cost-Effective Metal-Doped ZrO_2_ for Oxygen Evolution Reaction

**DOI:** 10.1007/s40820-024-01403-7

**Published:** 2024-04-25

**Authors:** Yuefeng Zhang, Tianyi Wang, Liang Mei, Ruijie Yang, Weiwei Guo, Hao Li, Zhiyuan Zeng

**Affiliations:** 1 Department of Materials Science and Engineering, and State Key Laboratory of Marine Pollution, City University of Hong Kong, 83 Tat Chee Avenue, Kowloon, 999077 Hong Kong People’s Republic of China; 2 Advanced Institute for Materials Research (WPI-AIMR), Tohoku University, Sendai, 980-8577 Japan; 3 Shenzhen Research Institute, City University of Hong Kong, Shenzhen, 518057 People’s Republic of China; 4 Shanxi Supercomputing Center, Lvliang, 033000 Shanxi People’s Republic of China

**Keywords:** Oxygen evolution reaction, Metal oxide, Electrocatalysis, Surface Pourbaix analysis, Doping

## Abstract

The design of cost-effective electrocatalysts is an open challenging for oxygen evolution reaction (OER) due to the “stable-or-active” dilemma. Zirconium dioxide (ZrO_2_), a versatile and low-cost material that can be stable under OER operating conditions, exhibits inherently poor OER activity from experimental observations. Herein, we doped a series of metal elements to regulate the ZrO_2_ catalytic activity in OER via spin-polarized density functional theory calculations with van der Waals interactions. Microkinetic modeling as a function of the OER activity descriptor (*G*_O*_-*G*_HO*_) displays that 16 metal dopants enable to enhance OER activities over a thermodynamically stable ZrO_2_ surface, among which Fe and Rh (in the form of single-atom dopant) reach the volcano peak (i.e. the optimal activity of OER under the potential of interest), indicating excellent OER performance. Free energy diagram calculations, density of states, and ab initio molecular dynamics simulations further showed that Fe and Rh are the effective dopants for ZrO_2_, leading to low OER overpotential, high conductivity, and good stability. Considering cost-effectiveness, single-atom Fe doped ZrO_2_ emerged as the most promising catalyst for OER. This finding offers a valuable perspective and reference for experimental researchers to design cost-effective catalysts for the industrial-scale OER production.

**Supplementary Information:**

The online version contains supplementary material available at 10.1007/s40820-024-01403-7.

## Highlights

- Surface energy and surface Pourbaix diagram reveal that ZrO_2_ ($\overline{1 }11$) is the most thermodynamically stable facet and is preferentially occupied by HO* at the equilibrium potential of oxygen evolution reaction (OER).
- Microkinetic modeling analyzed the OER activity of 40 single-metal doped ZrO_2_ and identified 16 metals exhibit improved catalytic activity, with Rh and Fe dopants showing the remarkable improvement.
- Thermodynamic free energy diagrams, density of states analysis, and ab initio molecular dynamics simulations further confirm that Fe–ZrO_2_ and Rh–ZrO_2_ are highly promising catalysts for OER, showcasing low Δ*G* for the rate-determining step, high conductivity, and exceptional stability.

**Supplementary Information:**

The online version contains supplementary material available at 10.1007/s40820-024-01403-7.

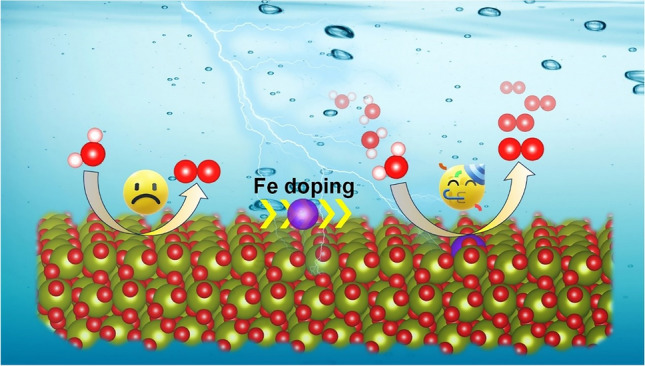

## Introduction

Proton exchange membrane water electrolysis offers an attractive means to store and convert renewable energy into sustainable hydrogen [1–3]. Water electrolysis comprises two half-reactions: oxygen evolution reaction (OER) and hydrogen evolution reaction (HER). Extensive studies have displayed that OER on the anode suffers from sluggish kinetics and poor stability resulting in a significant efficiency loss, thereby becoming the main obstacle for the practical implementation of electrochemical water splitting [4–11]. Therefore, it is vital to rationally search and design OER electrocatalysts with high activity and durability. Moreover, maintaining a delicate equilibrium between stability and activity poses a significant challenge for various electrocatalysts under OER operating conditions.

Zirconium dioxide (ZrO_2_), a highly sustainable material, has excellent thermal stability, making it highly desirable in various applications. For instance, nano-ZrO_2_ displayed remarkable aromatic selectivity and stability during a 120-h test [12]. ZrO_2_ also boasts exceptional hydrothermal stability during the CO_2_ methanation process under high temperatures and pressures [13]. Notwithstanding this, the OER activity of ZrO_2_ was known to be notably low [14–16]. Doping, a frequently utilized and remarkably efficient modulation strategy, can enhance the electrical conductivity and catalytic performance of materials [17–21]. For example, Mishra et al. demonstrated that optimal cobalt doping (5%) in CuO improved OER activity significantly with a low overpotential of 120 mV and a small charge transfer resistance of 2.58 Ω [22]. Additionally, Fu and co-workers demonstrated that Ce dopant induced electron redistribution of CoO to stabilize Co–O bonds and displayed an optimal binding strength with OER intermediates, manifesting favorable activity with an overpotential of 261 mV [23]. Therefore, it is possible to leverage the inherent stability of ZrO_2_ to conduct single-atom doping research, aiming to ensure its stability while enhancing its activity.

**Fig. 1:**
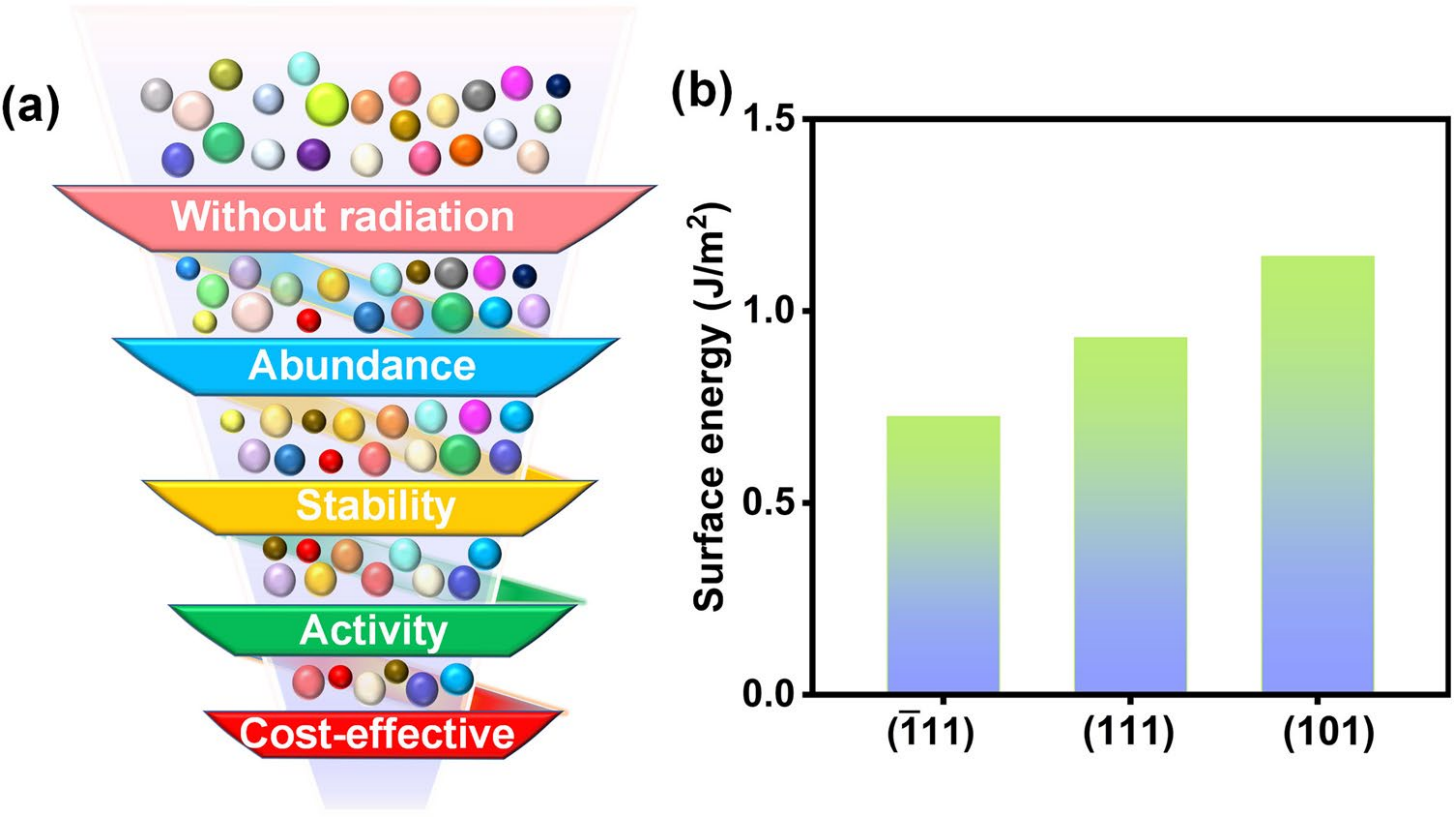
**a** Proposed strategy for screening metals capable of enhancing the OER activity of ZrO_2_ via doping. **b** Calculated surface energies of the three low-index slabs of monoclinic ZrO_2_. More details can be found in Fig. S1

Herein, we analyzed the electronic structure and OER performance of ZrO_2_ by doping a series of metals with flexible valence states, based on spin-polarized density functional theory calculations with van der Waals corrections (DFT-D3). First, we identified the thermodynamically stable ZrO_2_ surface, the ($\overline{1 }11$) surface, based on surface energy calculations with benchmarking with experimental literature. Subsequently, we analyzed the surface state of the identified stable ZrO_2_ surface via surface Pourbaix diagram calculations and found that the ZrO_2_ surface tends to be occupied by HO* under solution conditions at OER equilibrium potential. Finally, via a stratified catalyst design and screening strategy with various potential dopants for ZrO_2_ (Fig. 1a), we identified Fe as a promising dopant element by considering the effects of potential radiation, abundance, stability, activity, and cost-effectiveness. This work provides experimentalists with potentially superior catalysts worthy of future validation.

## Computational Details

The Vienna ab initio simulation package (VASP) was used to perform all spin-polarized DFT calculations [24, 25]. The generalized gradient approximation (GGA) in the form of revised Perdew–Burke–Ernzerhof (RPBE) [26] was employed to describe the exchange–correlation potentials. The Hubbard U parameter, representing the strong correlation repulsion energy between electrons with opposite spins, was added to the Fe (*U* = 4 eV) [27, 28], Co (*U* = 3 eV) [29, 30], Ni (*U* = 6.6 eV) [31], and Mn (*U* = 4 eV) [27, 32] 3*d* electrons. The electron–ion interaction was described with the projector-augmented wave method [33]. A plane-wave basis set of 480 eV was adopted. ZrO_2_ generally exists in three polymorphs [34–36]: monoclinic at room temperature, tetragonal at 1480–2650 K, and cubic at > 2650 K. Because the high thermodynamic stability is compulsory for conventional electrocatalysis under room temperature, herein, we further discuss the ZrO_2_ in a monoclinic form. Initial magnetic moments of 5, 0.6, 5, and 5 are set for the magnetic materials Fe, Co, Ni, and Mn, respectively. The zero-damping DFT-D3 method of Grimme was used to correct the van der Waals interactions [37]. The convergence criterion was set to be lower than 0.02 eV Å^−1^ for the force and 10^–6^ eV per atom for energy. A 2 × 2 × 1 Monkhorst–Pack was used for *k*-point sampling to describe the supercell [38]. A vacuum layer of 18 Å was set in the *z*-direction to avoid the interaction between periodic images. We employed ab initio molecular dynamics (AIMD) simulations in a canonical NVT ensemble to assess the thermodynamic stability of structures. The whole simulation lasted for 10 ps with a time step of 2 fs at 300 K. Furthermore, crystal orbital Hamilton population (COHP) was also calculated to analyze the local chemical bond properties in periodic systems. COHP is obtained via multiplying the Hamiltonian matrix by the corresponding DOS matrix. Combining it with the powerful LOBSTER tool [39], capable of handling plane-wave basis sets, allows for the visualization of COHP diagrams and analysis of bond strength.

The changes in Gibbs free energies for each elementary step were calculated using the following equation [40]:

1 $$\Delta G=\Delta E+\Delta {\text{ZPE}}-T\Delta S+\int {C}_{p}dT+ \Delta {G}_{pH}+{\Delta G}_{U}$$

where $\Delta E$, $\Delta {\text{ZPE}}$, $\Delta S$, and ${C}_{p}$ are the changes in the electronic energy directly obtained from DFT, zero-point energy, entropy, and heat capacity, respectively. *T* is the temperature (298.15 K). The entropies of molecules in the gas phase were acquired from the NIST database. $\Delta {G}_{pH}$ is the free energy contribution due to the variations in H concentration, expressed by the Nernst equation as $\Delta {G}_{pH}=2.303{k}_{B}T{\text{pH}}$. In this work, we set pH to zero. ${\Delta G}_{U }=-neU$, where n was the electron transfer number and U was the applied potential.

Under the acidic condition, the thermodynamic potential for the oxidation of H_2_O to produce O_2_ involving 4 electron transfers is described as:

2 $$2{H}_{2}O \to {O}_{2}+4{H}^{+}+4{e}^{-}$$

The thermodynamic overpotential (*η*) is the important indicator to judge the catalytic activities of a catalyst, which can be obtained by using the following equation:

3 $$\eta = \frac{{\Delta G}_{max}}{e}-1.23 V$$

where ${\Delta G}_{max}$ is the maximum free energy change among the four elementary steps, and 1.23 V is the equilibrium potential. A lower $\eta$ implies higher catalytic activity, and the $\eta$ of an ideal catalyst is zero.

The surface energy is the energy required to break intermolecular chemical bonds when creating a new surface, expressed by the formula [41, 42]:

4 $${\gamma }_{s}=\frac{\left[{E}_{slab-unrelax}-N{E}_{bulk}\right]}{2A}+\frac{\left[{E}_{slab-relax}-{E}_{slab-unrelax}\right]}{A}$$

where ${E}_{slab-unrelax}$ and ${E}_{slab-relax}$ is the total energy of the unrelaxed and relaxed slab, respectively. ${E}_{bulk}$ is the bulk energy per atom, *N* is the number of atoms in the slab, and *A* is the surface area. The kinetic volcano model was derived based on the potential-dependent kinetic information as a function of current density and adsorption free energies of HO* and O*, with the details and parameters shown in Ref. [43].

The surface Pourbaix diagram was built based on the computational hydrogen electrode (CHE) method [40], which offers thermodynamic insights into the water activation-induced surface coverage of an electrocatalyst under operating conditions [44–46], expressed as follows:

5 $${H}_{n}{O}_{m}^{*}+\left(2m-n\right)\left({e}^{-} {+ H}^{+}\right)\rightleftarrows *+{ mH}_{2}O$$

where *m* and *n* are the number of oxygen and hydrogen atoms of the adsorbate, respectively. The differences in free energy were calculated using:

6 $$\Delta G ={G}_{bare}+m{G}_{{H}_{2}O}-{G}_{total}-(2m-n)\left(\frac{1}{2}{H}_{2}-{U}_{SHE}-2.303{k}_{B}T*pH\right)$$

Here, ${U}_{SHE}$ refers to the potential relative to the standard hydrogen electrode (SHE), and ${k}_{B}$ is the Boltzmann constant (8.617343 × 10^–5^ eV K^−1^). The surface Pourbaix diagrams were modeled with the *CatMath* online platform through cloud computing [47].

## Results and Discussion

### Surface Energy and Surface Pourbaix Analysis

The most stable phase of ZrO_2_ at low temperature is monoclinic [36] taking the P2_1_/c space group, and the optimized lattice parameters (*a* = 5.15 Å, *b* = 5.26 Å, *c* = 5.30 Å) closely match the experimental values [48]. To determine the most stable exposed facet, we conducted surface energy calculations on three representative low-index planes, namely ($\overline{1 }11$), (111), and (101), all of which are intricate models featuring O-terminated, Zr-terminated, and Zr and O co-terminated interfaces. Figure S1 provides a visual representation of all possible surface structures maintaining a stoichiometric ratio consistent with the bulk, along with their corresponding surface energy values. A lower surface energy indicates a higher propensity for the formation of chemical bonds, thus suggesting a thermodynamic preference for crystal growth along that specific plane. The minimum surface energy values for ($\overline{1 }11$), (111), and (101) planes are 0.725, 0.930, and 1.142 J m^−2^, respectively (Fig. 1b). This implies that ZrO_2_ ($\overline{1 }11$) is the most thermodynamically stable exposed facet, aligning consistently with earlier theoretical prediction and experimental observation [49, 50]. This finding identifies an important substrate model, ZrO_2_ ($\overline{1 }11$), which is a prerequisite for OER activity analysis.

**Fig. 2:**
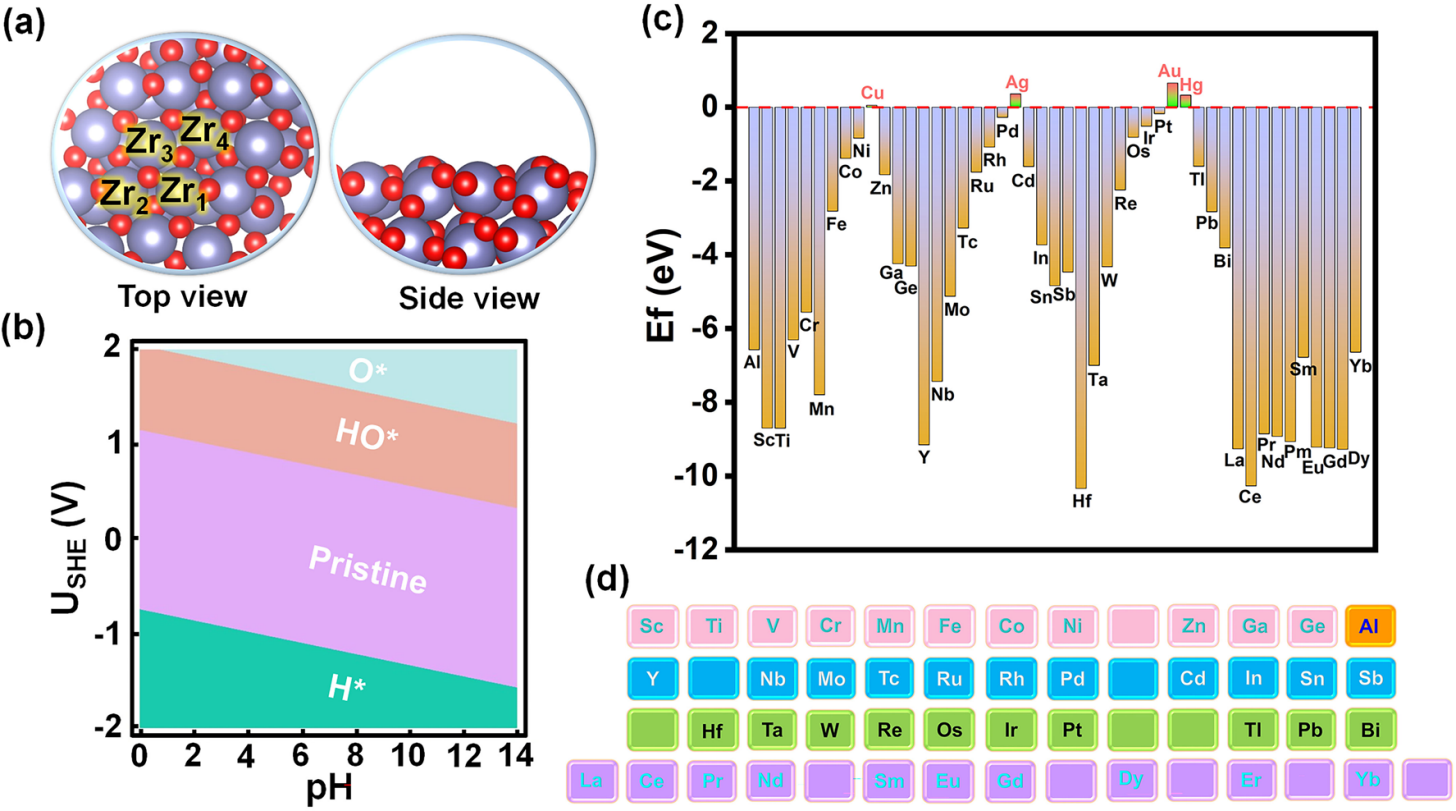
**a** Optimized configuration of ZrO_2_ with top and side views. The purple and red spheres represent Zr and O, respectively. **b** Calculated surface Pourbaix diagram of the ZrO_2_ surface. **c** Formation energy values of different single-metal atoms doped ZrO_2_. **d** The feasible metals for doping on the surface of ZrO_2_.

As Fig. 2a illustrated, O-terminated ZrO_2_ configuration holds many various adsorption sites. H*, O*, or HO* species generated through water activation are possible to pre-covered on the surface of catalysts under OER operating conditions, which renders the oxide system may deviate from its pristine stoichiometric form [43] and exerts substantial influences on reaction overpotential. Surface Pourbaix analysis can provide a valuable approximation of the real state under OER conditions [44–46]; thus, we calculated the surface Pourbaix diagram (Fig. 2b). The results illustrate that the surface of ZrO_2_ will be initially occupied by HO* at *U*_SHE_ > 1.13 V *vs*. reversible hydrogen electrode (RHE), which is lower than the equilibrium potential of OER (1.23 V vs. RHE); that means, under working conditions, the surface is no longer in its pristine state but instead adopts a restructured configuration enriched with HO*. However, given that HO* also serves as the initial adsorption intermediate of OER, we can directly use the original model to analyze the OER activity. Surface Pourbaix diagram provides a detailed analysis of the surface occupation states to help us obtain a theoretical substrate model that is closer to the experiment. To assess the viability of doping various metals onto the ZrO_2_ surface, we calculated the doping formation energy (Fig. 2c). The most stable doping configurations for each metal are shown in Figs. S2 and S3. Generally, the doping formation energy is calculated to assess the thermodynamic stability of catalysts, wherein the negative value indicates the high stability and vice versa. As Fig. 2c shows, Cu, Ag, Au, and Hg dopants exhibit positive formation energies, which means these dopants are difficult to replace Zr and fix on the ZrO_2_ stably. Conversely, the remaining metal dopants display negative formation energies, indicating they are thermodynamical feasibility to substitute Zr and form stable catalysts. All feasible metal elements for doping are listed in Fig. 2d, which are subsequently investigated for the OER analysis. Specifically, metals are arranged in the order of the periodic table of elements except Al. The yellow, pink, blue, green, and purple backgrounds correspond to elements from the third, fourth, fifth, sixth periods, and rare earth elements, respectively. This order can identify elements in empty positions, which are subsequently disregarded.

### OER Activity Screening Based on the Volcano Activity Model

**Fig. 3:**
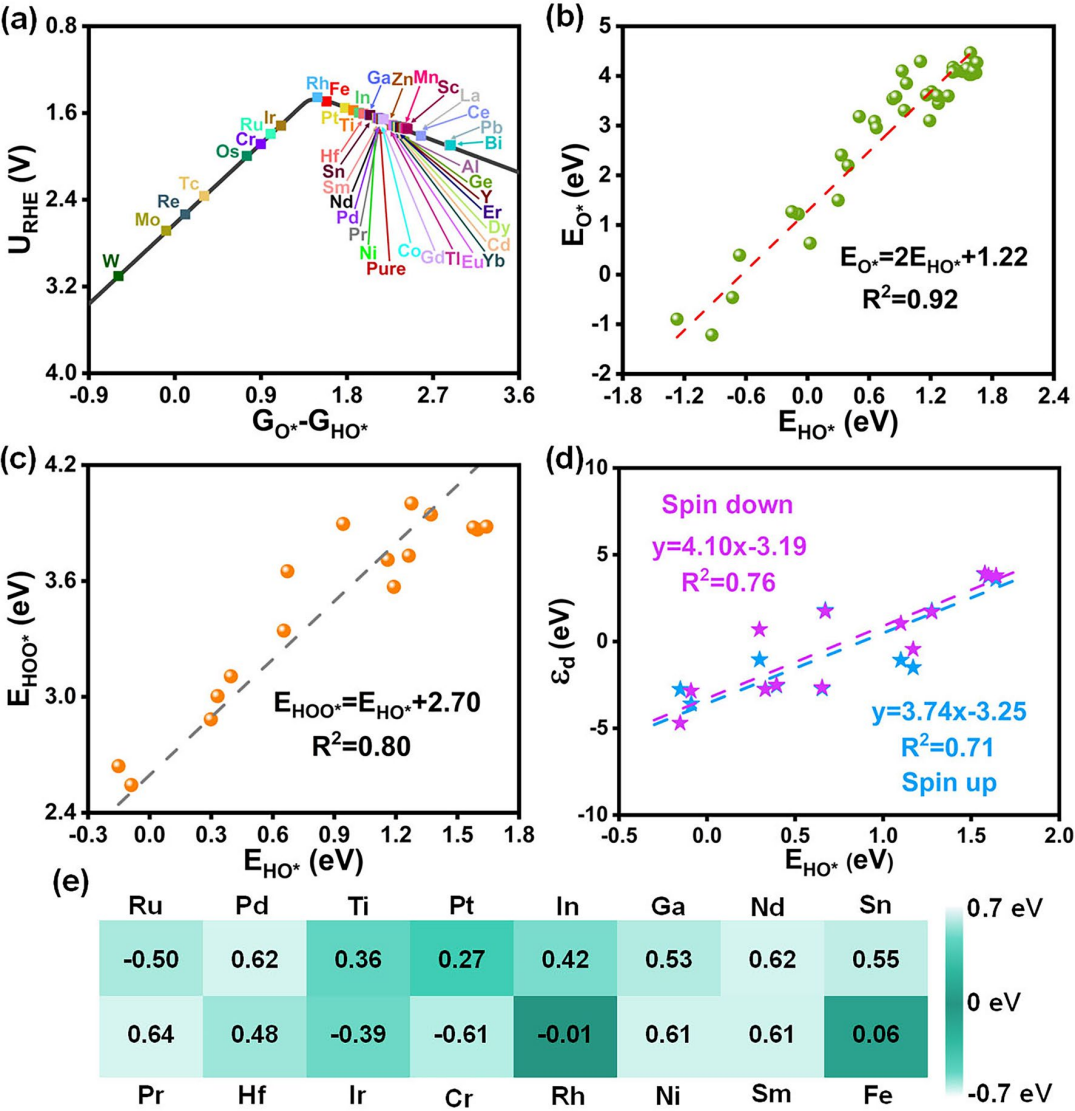
**a** Kinetic OER activity volcano model as a function of *G*_O*_-*G*_HO*_ at 5 $\mu$ A cm^−2^ (black line). **b** Scaling relation between *E*_O*_ and *E*_HO*_ on feasible metals doped ZrO_2_. The linear scaling relations of *E*_HO*_ vs. **c** *E*_HOO*_ and **d** ${\varepsilon }_{d}$ on elevated metal doped ZrO_2_. **e** A heatmap represents the magnitude of values as a color. Values (unit eV) were obtained by *G*_O*_-*G*_HO*_ = 1.5, and darker colors correspond to lower values implying higher activity.

The *G*_O*_-*G*_HO*_ is generally regarded as a crucial descriptor for evaluating OER activity since it quantifies the capacity of surface oxygen atoms to make and break bonds with hydrogen and oxygen atoms, which is prone to be the most thermodynamically challenging reaction steps for OER [2, 43, 51]. To achieve more accurate predictions, we employed the microkinetic modeling as the function of *G*_O*_-*G*_HO*_ and potential at various currents [43, 52]. The current curves were obtained by numerically solving the rate of HOO formation considering the O $\to$ HOO transition state. Figure 3a illustrates the kinetic OER activity relating potential to *G*_O*_-*G*_HO*_ at a current density of 5 $\mu$ A cm^−2^, featuring the characteristic volcano-shape. On the left side of the peak, decreased *G*_O*_-*G*_HO*_ values correspond to lower OER kinetic activity. Similarly, catalysts exhibit the decaying OER performance along the right arm of the volcano curve. Catalysts close to volcanic peaks, such as Co, Mn, Rh, Fe, and Pt, demonstrate good catalytic activity. Especially, Rh sitting at the volcano peak, with an optimal *G*_O*_-*G*_HO*_ value, requires relatively low overpotentials to achieve high reaction rates in acidic solutions, demonstrating the highest OER activity. In addition, we plot potential as a function of *G*_O*_-*G*_HO*_ at 1 mA cm^−2^ (Fig. S4) and 1 A cm^−2^ (Fig. S5), and the results display that the activity order may change as the current increases, but the qualitative order remains constant; that is, a good catalyst is always good at different currents. Figure 3b displays a linear scaling relation between the adsorption energy of O* (*E*_O*_) and HO* (E_HO*_), with the identified slope and intercept close to those of higher-index (where *h*^2^ + *k*^2^ + *l*^2^ > *1*) metal oxide surfaces [52]. Figure 3c further demonstrates that the adsorption energy of HOO* (*E*_HOO*_) and E_HO*_ exists a good linear correlation and a constant difference between E_HOO*_ and E_HO*_ on effectively doped catalysts, which implies *G*_HOO*_ – *G*_HO*_ = (*G*_O*_ – *G*_HO*_) + (*G*_HOO*_ – *G*_O*_) = constant, i.e., *G*_HOO*_ – *G*_HO*_ = (*G*_O*_ – *G*_HO*_) + [constant(*G*_O*_ – *G*_HO*_)]. Therefore, *G*_O*_ – *G*_HO*_ serves as a distinctive descriptor elucidating the OER activity, further reinforcing the rationale behind the selection of Fig. 3a. Furthermore, E_HO*_ and ${\varepsilon }_{d}$-up/${\varepsilon }_{d}$-down demonstrate a robust linear correlation, as depicted in Fig. 3d. Such correlation offers valuable insights into finding optimal HO binding energies by manipulating the ${\varepsilon }_{d}$ value. Generally, the optimal OER catalyst has surface hydroxide deprotonation free energy value of 1.5 to 1.7 eV [43, 53–55]. In our work, taking 1.5 eV as the standard fits well with the dynamic trend (Fig. 3a), we use (*G*_O* _− *G*_HO*_ = 1.5 eV) as the descriptor to further embody catalytic behavior. A smaller absolute value of (*G*_O* _− *G*_HO*_ = 1.5 eV), represented by a darker color, suggests better OER performance (Fig. 3e). Figure 3e selects all metals closing to the volcanic peak exhibits improved OER performance compared to the pristine ZrO_2_, in which Rh has the best catalytic activity with values closest to zero. Theoretical overpotential (*η*), a fundamental thermodynamic parameter, has emerged as a crucial metric for evaluating and predicting the OER performance of catalysts. Thus, we plot *η* as a function of *G*_O*_ − *G*_HO*_, which has a volcano relationship (Fig. S6). Rh standing on the peak of the volcano map exhibits the highest OER activity with minimum *η*, followed by Fe, Pt, etc. This thermodynamic volcano diagram trend is roughly consistent with the dynamic volcano diagram (Fig. 3a), and the gap may be attributed to the omission of transition states in thermodynamics. To acquire a more profound comprehension of the impact of doping on the electronic properties of the catalyst, we calculate the density of states (DOS) of ZrO_2_ (Fig. S7a). Near the Fermi level, the valence band is predominantly contributed by O orbitals, while the conduction band is primarily composed of Zr orbitals. A wide forbidden region exists between the top of the valence band and the bottom of the conduction band with a bandgap of 2.6 eV, further observed by the band structure diagram (Fig. S7c). Also, the precise HSE functional displayed that ZrO_2_ is a semiconductor material with a wide bandgap of 4.45 eV (Fig. S7b). Figures S8 and S9 show the DOS of metal doped ZrO_2_. The bandgap values exhibit a progressively decreasing trend in the following order: Ir (2.54 eV), Ru (2.49 eV), Sn (2.28 eV), Ti (1.82 eV), and Cr (1.71 eV). In, Sm, Pr, Fe, Nd, Ni, Pd, Ga, Co, Pt, Mn, and Rh metals, on the other hand, induce a direct transformation of the catalyst from a semiconductor to a semi-metallic property characterized by a band gap of 0 eV, effectively promoting the electron transfer and thereby improving the conductivity of the ZrO_2_. In short, the microkinetic volcano diagrams screen out some promising catalysts, the linear relationship obtains the abnormal adsorption points and the correlation between different adsorbates, and DOS uncover enhanced conductivity of catalysts through doping.

### Free Energy Analysis

**Fig. 4:**
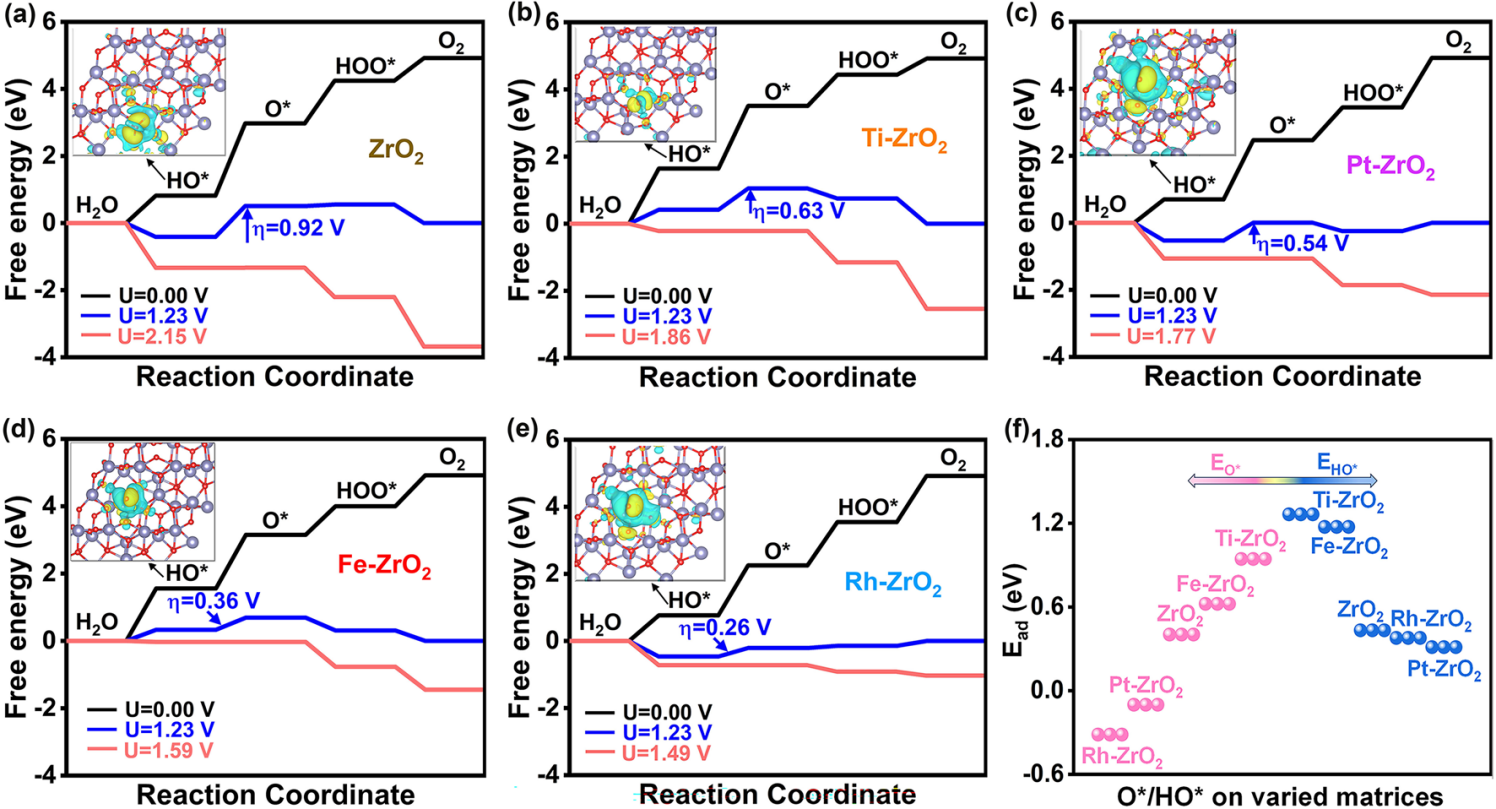
Gibbs Free energy diagram of OER on **a** ZrO_2_, **b** Ti–ZrO_2_, **c** Pt–ZrO_2_, **d** Fe–ZrO_2_, and **e** Rh–ZrO_2_. **f** Adsorption energy values of O* (pink line) and HO* (blue line) on five varied substrates. The inset is the charge density difference of HO*, and the isosurface value was set to 0.001 e Å^−3^

Gibbs free energy diagrams were established to visualize the OER intermediate formations of four doped metals closest to the volcanic peak and with stable intermediates. Figure 4a exhibits the free energy diagram of OER on ZrO_2_. The first step, the formation of HO*, is thermodynamically feasible. Then, HO* dissociates into H^+^ and O*, which is thermodynamically challenging with a higher onset potential of 2.15 V. The next proton-electron transfer steps of HOO* formation and the generation of O_2_ in the last step are energetically supported. Therefore, HO* deprotonation to generate O* on ZrO_2_ is the most challenging step, which is the potential determining steps (PDS), requiring a *η* of 0.92 V to make all steps thermodynamically downhill (watermelon red line). Similarly, HO*$\to$ O* is the PDS of Ti–ZrO_2_ with a *η* of 0.63 V, Pt–ZrO_2_ with a *η* of 0.54 V, Fe–ZrO_2_ with a *η* of 0.36 V and Rh–ZrO_2_ with a *η* of 0.26 V (Fig. 4b–e), which aligns closely with the microkinetic volcano diagram trend (Fig. 3a). The specific adsorption energies of O* and HO* are calculated in Fig. 4f. The order of adsorption strength for O* is Rh–ZrO_2_ > Pt–ZrO_2_ > ZrO_2_ > Fe–ZrO_2_ > Ti–ZrO_2_, and that of HO* is Pt–ZrO_2_ > Rh–ZrO_2_ > ZrO_2_ > Fe–ZrO_2_ > Ti–ZrO_2_, which is further explained by the charge density difference map (Fig. S10 and inset in Fig. 4). In short, the free energy diagram reveals that Fe–ZrO_2_ and Rh–ZrO_2_ are highly active catalysts with only slightly higher $\Delta$*G*.

For the comparison of different adsorbate species, we relied on thermodynamic analysis, while the dynamic factor was lacking. Therefore, we performed AIMD simulations on Rh–ZrO_2_ with different species based on its optimal catalytic activity (Fig. S11a–c). The results show that they are thermodynamically stable. In addition, we also evaluated the thermal stability of ZrO_2_, Fe–ZrO_2_, and Rh–ZrO_2_ (Fig. S11d–f). The results demonstrate that these systems maintain their structural integrity, in which energy and temperature oscillate around equilibrium within thermal perturbations. Thus, they remain thermodynamically stable and catalytic activity for extended duration.

### Interatomic Bond Strength Analysis

**Fig. 5:**
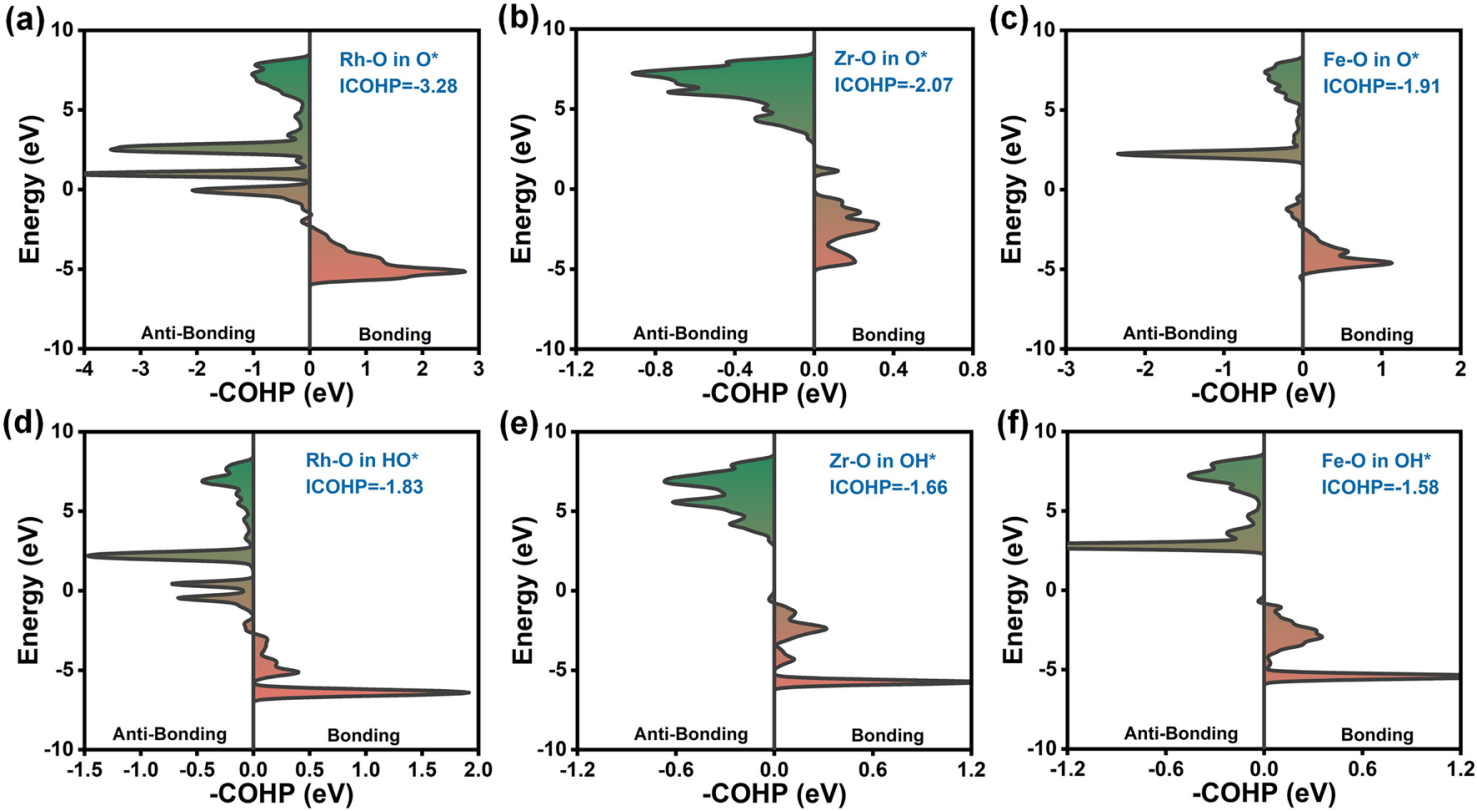
Crystal orbital Hamilton population (COHP) analysis of the interactions between **a** Rh–O in O*, **b** Zr–O in O*, **c** Fe–O in O*, **d** Rh–O in HO*, **f** Zr–O in HO*, and **e** Fe–O in HO*

COHP can provide insights into the strength and nature of chemical bonds between atoms. Integrated COHP (ICOHP) is a quantitative method for measuring chemical bond strength, and a more negative ICOHP value indicates a stronger bond strength between atoms. As Fig. 5 exhibited, we calculate the COHP and ICOHP of metal-O (O* and HO*) bond to deeply understand the bonding mechanism. The sequence of bonding strength with O* is Rh (− 3.28 eV) > Zr (− 2.07 eV) > Fe (− 1.91 eV) (Fig. 5a–c), and with O in HO* is Rh (− 1.83 eV) > Zr (− 1.66 eV) > Fe (− 1.58 eV) (Fig. 5d–f). The ICOHP values explain the adsorption energy trend well. The strong bonding interaction between Rh and O* and HO* results in a lower energy requirement for stabilizing the OER intermediates, which is the key reason of its optimal catalytic performance. Bader charge and D band center (${\varepsilon }_{d}$) are also further quantified (Fig. S12). Compared with Fe dopant, Rh loses more charge to O* and HO* (Fig. S12a) and has a smaller negative ${\varepsilon }_{d}$ value (Fig. S12b, c), indicating underlying reasons for the strong adsorption capacity of Rh.

Based on comprehensive analysis, both Rh–ZrO_2_ and Fe–ZrO_2_ are potential candidates for enhancing OER efficiency. The Rh doping exhibits a lower overpotential, but its shortage limits industrial application. In contrast, Fe, a cost-effective and readily available metal, has not only been successfully doped into ZrO_2_ in experiments [56–58] but also regraded as an ideal dopant for enhancing OER activity [59, 60]. Therefore, Fe holds great promise as a modulating agent for industrial-scale OER application.

## Conclusion

In summary, we have analyzed the effects of 40 different single-metal doping to regulate the OER activity of ZrO_2_ using a stratified screening process based on spin-polarized DFT-D3, surface Pourbaix analysis, microkinetic modeling, cost-effectiveness analysis, and bonding analysis. We identified 16 metals exhibit improved catalytic activity, with Rh and Fe dopants showing the remarkable improvement. These doped metals reduce the band gap of ZrO_2_, thereby significantly increasing conductivity. In addition, the thermodynamic free energy diagram shows that the theoretical overpotential of Ti, Pt, Fe, and Rh metals are 0.63, 0.54, 0.36, and 0.26 V, respectively, which aligns well with the kinetic volcano model. Compared with Fe dopant, the smaller negative ${\varepsilon }_{d}$ value facilitates Rh dopant to stabilize the OER intermediates well and thus exhibits higher catalytic activity, but its high cost and shortage limit industrial-scale application. Therefore, the cost-effectiveness Fe-ZrO_2_ catalyst holds more promise and practicality. This finding provides valuable insights into the design and development of stable, low-cost, and high-performance OER catalysts for commercial-scale water splitting.

## Supplementary Information

Below is the link to the electronic supplementary material.

Supplementary file1 (PDF 2832 KB)
