# Supplementary material for: Rational Design of Cost-Effective Metal-Doped ZrO2 for Oxygen Evolution Reaction
Source: Nanomicro Lett. 2024 Apr 25;16:180. doi: 10.1007/s40820-024-01403-7 (PMC11045712; doi:10.1007/s40820-024-01403-7)
Supplement: Supplementary file 1 — Supplementary file1 (PDF 2832 KB) [file 40820_2024_1403_MOESM1_ESM.pdf]

Supporting Information for

Rational Design of Cost-Effective Metal-Doped ZrO_2 for Oxygen Evolution Reaction

Yuefeng Zhang[1], Tianyi Wang[2], Liang Mei[1], Ruijie Yang[1], Weiwei Guo[4], Hao Li[2,]*, and Zhiyuan Zeng[1, 3,]*

[1] Department of Materials Science and Engineering, and State Key Laboratory of Marine Pollution, City University of Hong Kong, 83 Tat Chee Avenue, Kowloon, Hong Kong 999077, P. R. China

[2] Advanced Institute for Materials Research (WPI-AIMR), Tohoku University, Sendai 980-8577, Japan

[3] Shenzhen Research Institute, City University of Hong Kong, Shenzhen 518057, P. R. China

[4] Shanxi Supercomputing Center, Lvliang, Shanxi Province 033000, P. R. China

* Corresponding authors. E-mail: li.hao.b8@tohoku.ac.jp (Hao Li); zhiyzeng@cityu.edu.hk (Zhiyuan Zeng)

Supplementary Figures

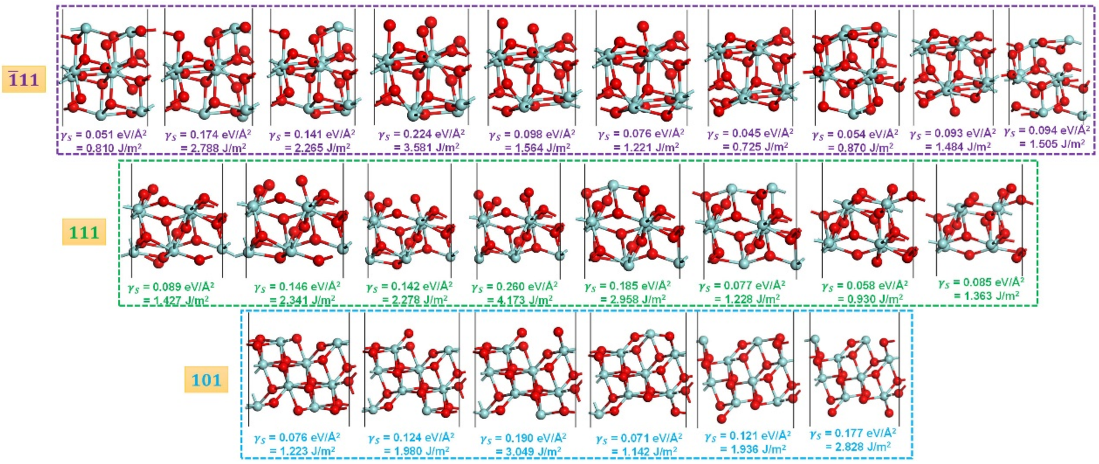

Fig. S1 The surface structures and corresponding surface energy values of ZrO_2 ($\bar{1}11$), (111), and (101) slabs. Zr and O atoms are denoted by cyan and red balls, respectively

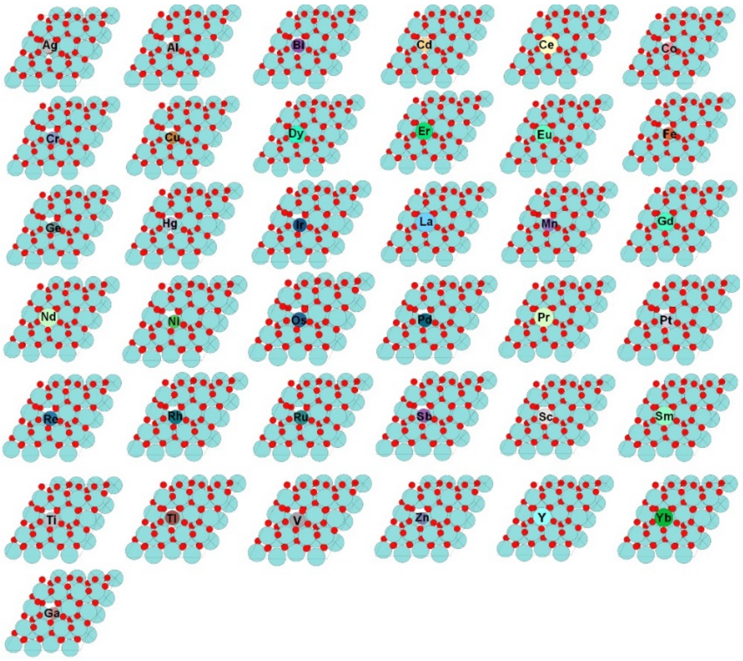

Fig. S2 Structural models corresponding to the metals whose most stable replacement site is the Zr3 site

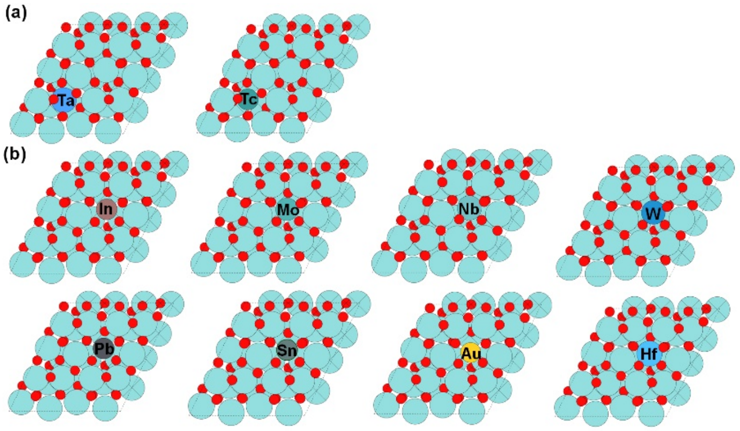

Fig. S3 Structural models corresponding to the metals whose most stable replacement site is the Zr2 site (**a**) and Zr4 site (**b**)

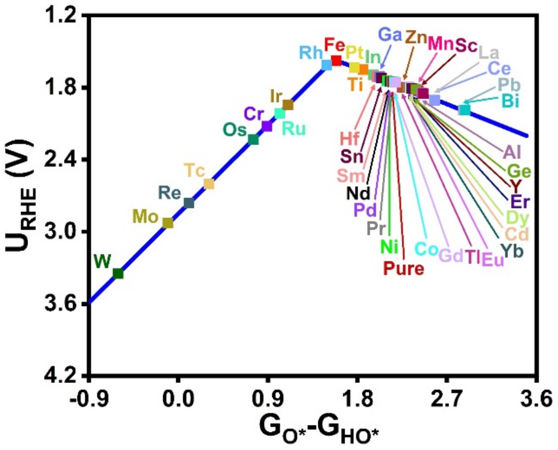

Fig. S4 Kinetic oxygen evolution reaction (OER) activity volcano plot as a function of G_{O*}-G_{HO*} at 1 mA/cm^2 (blue line)

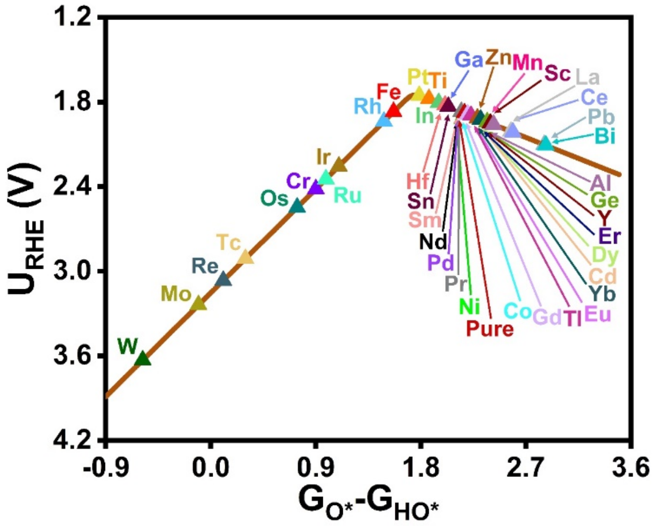

Fig. S5 Kinetic OER activity volcano plot as a function of G_{O*}-G_{HO*} at 1 A/cm^2 (saddle brown line)

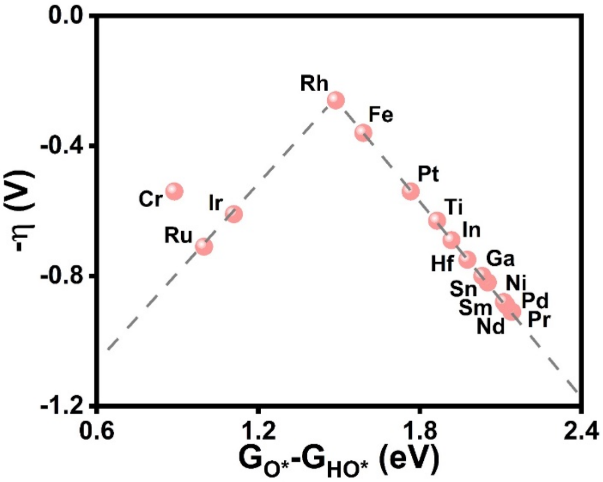

Fig. S6 Negative theoretical overpotential (η) is plotted as a function of the free energy difference between O* and HO* (G_{O*}-G_{HO*})

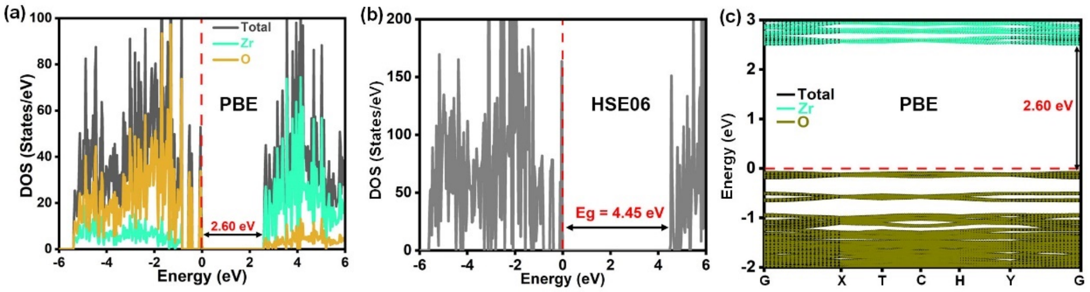

Fig. S7 Density of states (DOS) obtained through PBE functional (**a**) and HSE06 functional (**b**), and band structure (**c**) of ZrO_2 surface. The red dotted line corresponds to the Fermi level

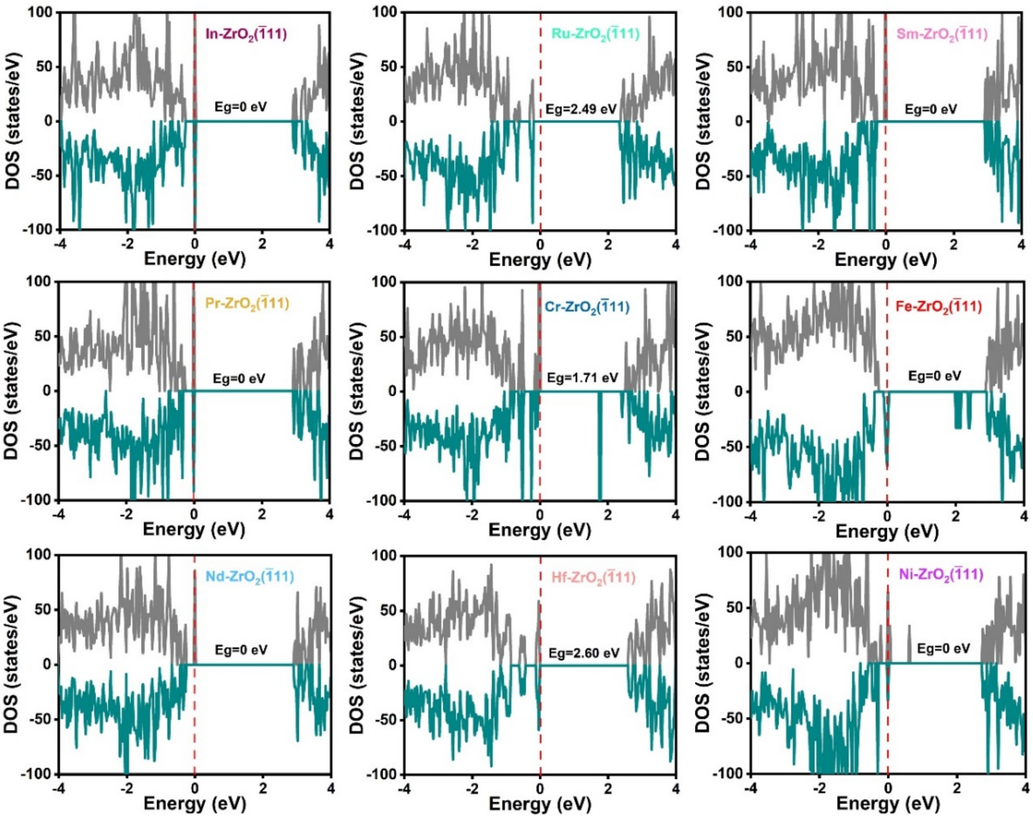

Fig. S8 DOS of the M-ZrO_2 (M=In, Ru, Sm, Pr, Cr, Fe, Nd, Hf, Ni). The gray line represents the spin-up orbit, and the green line represents the spin-down orbit

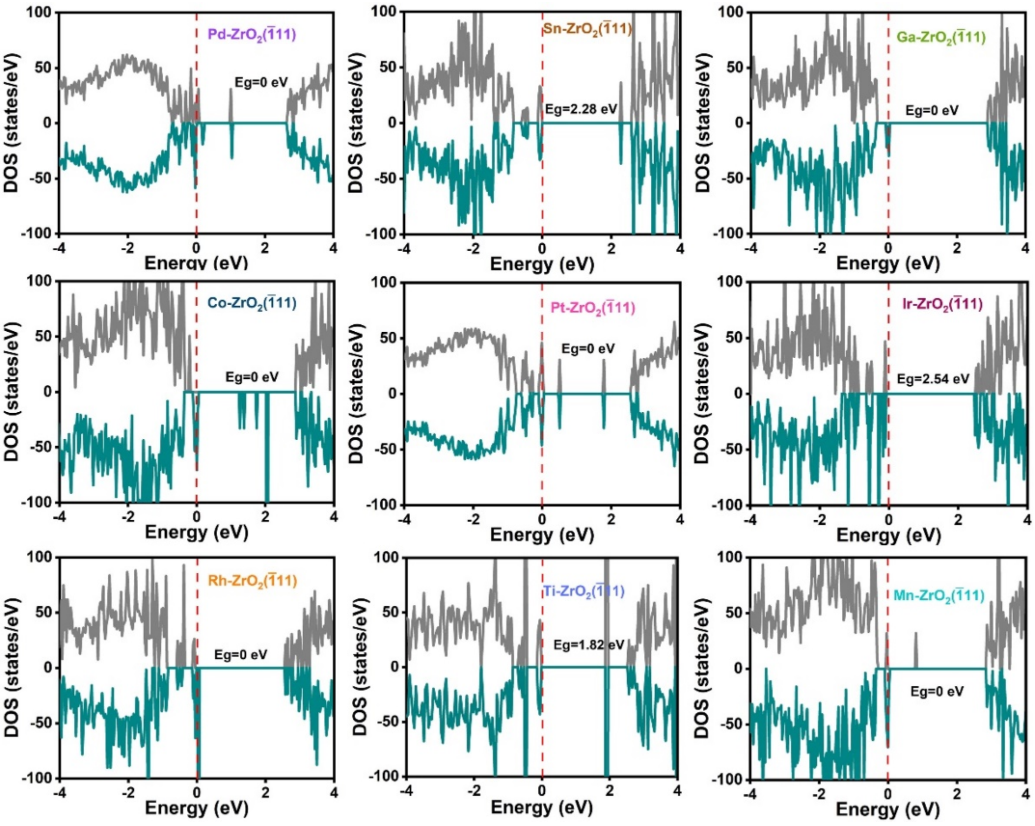

Fig. S9 DOS of the M-ZrO_2 (M=Pd, Sn, Ga, Co, Pt, Ir, Rh, Ti, and Mn). The gray line represents the spin-up orbit, and the green line represents the spin-down orbit

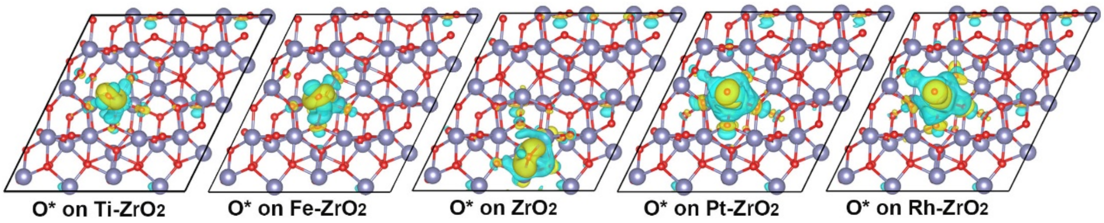

Fig. S10 Charge density difference of O* on Ti-ZrO_2, Fe-ZrO_2, ZrO_2, Pt-ZrO_2, and Rh-ZrO_2, respectively. The isosurface value was set to 0.001 e/Å^3

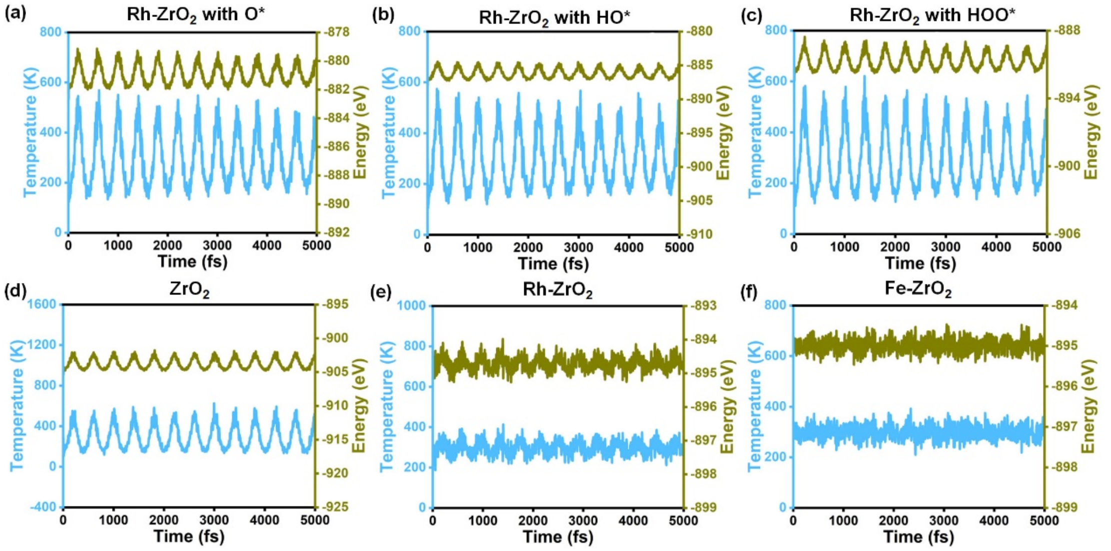

Fig. S11 *Ab initio* molecular dynamics (AIMD) simulations under 300 K for (**a**) ZrO_2, (**b**) Rh-ZrO_2, (**c**) Fe-ZrO_2, (**d**) Rh-ZrO_2 with O*, (**e**) Rh-ZrO_2 with HO*, and (**f**) Rh-ZrO_2 with HOO*

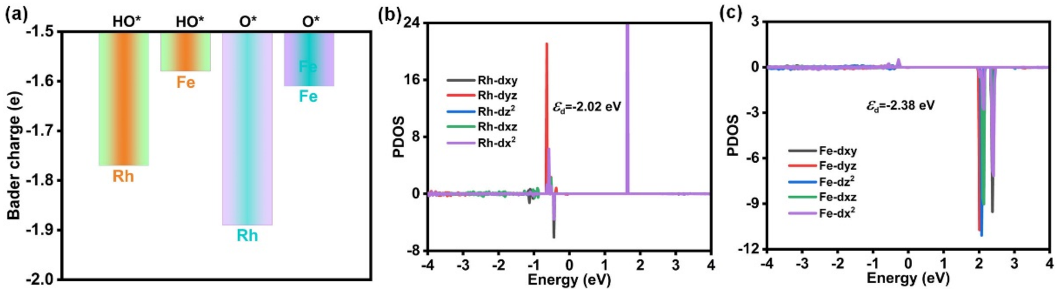

Fig. S12 (**a**) Charge changes produced by metals (Fe, Rh) binding to O* and HO*. D band center (ε_d) of (**b**) Rh-ZrO_2 and (**c**) Fe-ZrO_2
